# Locating automated external defibrillators in urban areas: considering different travel modes

**DOI:** 10.3389/fpubh.2026.1936570

**Published:** 2026-10-14

**Authors:** Weicong Luo, Jinhao Liu, Yuhan He, Fei Wang

**Affiliations:** 1 College of Public Administration, Huazhong Agricultural University, Wuhan, Hubei, China; 2 Wuhan Emergency Medical Center, Wuhan, China

**Keywords:** accessibility, AED, GIS, spatial optimization, travel modes

## Abstract

**Introduction:**

Out-of-hospital cardiac arrest (OHCA) is a time-critical emergency in which rapid access to automated external defibrillators (AEDs) can improve survival. However, most AED location studies assume a single travel mode and often use population alone as a proxy for demand. This study investigates how travel-mode assumptions affect AED accessibility and optimal deployment and develops a risk-informed multimodal spatial optimization framework.

**Methods:**

Using Wuhan, China, as a case study, an age-risk-weighted potential OHCA demand surface was constructed from 17,842 ambulance dispatch records and age-stratified population data across 1,161 1-km grid cells. The existing 3,569 AEDs were treated as fixed facilities. Accessibility was evaluated under driving, cycling, walking, Drive + Walk, and Cycling + Walk scenarios using a 4-min response threshold. Multimodal extensions of the Location Set Covering Problem (LSCP) and Maximal Covering Location Problem (MCLP) were applied to identify full-coverage requirements and priority locations.

**Results:**

Baseline demand-weighted coverage varied substantially by travel mode: 85.07% for driving, 68.27% for cycling, 31.55% for walking, 53.65% for Drive + Walk, and 45.41% for Cycling + Walk. The additional AEDs required for universal coverage ranged from 68 under driving to 521 under walking, with 327 required under Drive + Walk. Under the first-phase MCLP strategy, 65 new AEDs increased Drive + Walk demand-weighted coverage to 73.31%. Sensitivity analysis confirmed that the main findings were robust to alternative mode weights.

**Discussion:**

AED accessibility and optimal deployment are highly sensitive to responder mobility assumptions. Incorporating heterogeneous travel modes and age-specific OHCA risk can support more realistic, spatially targeted, and resource-efficient AED planning.

## 1 Introduction

Out-of-hospital cardiac arrest (OHCA) represents a major public health emergency, defined by the abrupt cessation of cardiac activity occurring outside a hospital setting. Survival from OHCA is highly time-sensitive, with the effectiveness of resuscitation declining rapidly as treatment is delayed. For shockable rhythms such as ventricular fibrillation, early defibrillation is the most decisive life-saving intervention. Evidence indicates that defibrillation administered immediately after witnessed ventricular fibrillation can yield survival rates exceeding 90%; however, survival decreases by approximately 7%−10% for each minute of delay (1, 2). In this context, automated external defibrillators (AEDs)—portable medical devices designed to analyze cardiac rhythms and deliver an electric shock when indicated—play a pivotal role in improving OHCA outcomes. As a core component of the “chain of survival,” AEDs enable rapid defibrillation by lay responders prior to the arrival of emergency medical services. Although studies have shown that cardiopulmonary resuscitation (CPR) combined with AED use within 3–5 min can achieve survival rates of 50%−70%, timely access to AEDs remains a major constraint in real-world rescue scenarios (3).

The increasing frequency of public health emergencies, together with demographic shifts such as population aging, has substantially elevated societal demand for AEDs, driving rapid expansion of locating those facilities in cities. In the United States, a pioneer in public defibrillation, legislation mandates AED deployment in key public locations like airports and stadiums, particularly in areas where emergency services cannot arrive within 5 min (4). The Japan national government has launched a National Public Access Defibrillation program, achieving AED coverage exceeding 80% in major public spaces such as train stations and schools (5). In the United Kingdom, national guidelines emphasize spatial accessibility, recommending that AEDs in public areas be located within an approximate walking distance of 300 m to enable timely bystander defibrillation (6). As one of the world's most populous countries and one facing pronounced population aging, China has an especially urgent public health need for AEDs. In recent years, multiple Chinese cities have increasingly incorporated time-sensitive AED access into local emergency planning and deployment guidance. In Wuhan, municipal policy requires AEDs to be provided in densely populated public places, while official municipal materials emphasize the “golden 4 min” for cardiac-arrest intervention (7, 8). Hangzhou's official AED configuration specification recommends that AEDs be obtainable and brought to the scene within 3–5 min (9), while Han et al. (10) used a 4-min response standard in a Nanjing pre-hospital facility study. Together, these practices highlight the time-space dimension of AED accessibility and support the use of a short time-based threshold for planning analyses.

Spatial optimization models are commonly used to improve AED accessibility by identifying where limited devices should be placed (or relocated) so that more OHCAs fall within a practical bystander retrieval distance and time window. Typically, these models combine historical OHCA locations (or risk surfaces) with a set of candidate sites. Coverage-based models include the Location Set Covering Problem (LSCP), which minimizes the number of facilities required to cover all demand within a predefined threshold (11), and the Maximal Covering Location Problem (MCLP), which maximizes covered demand for a fixed number of facilities (12). In contrast, accessibility-based formulations such as the p-median problem minimize weighted travel distance or time between demand points and facilities (13). Both types of models have been widely applied in studies on AED deployment [e.g., Tierney et al. (14), Bonnet et al. (15), Chen et al. (16)].

However, existing research on AED spatial optimization exhibits a major limitation. Most studies are based on a single transportation mode, such as walking or running, or vehicle travel (17–19). Multimodal healthcare accessibility has been examined in prior studies (20, 21), while Guo et al. (22) considered emergency-facility location on a multimodal transport network. These studies provide methodological precedents in adjacent healthcare-accessibility and emergency-facility planning, but they do not address AED location-allocation specifically. AED siting studies remain predominantly single-mode; therefore, this study compares walking, cycling, driving, and weighted multimodal accessibility scenarios within a unified optimization framework. In real-world urban emergency response scenarios—particularly in congested city centers or areas with poor street-network connectivity—the travel modes used by first responders to reach AED locations are highly diverse. Responses may involve walking directly to the scene, using shared bicycles, or, when conditions permit, traveling by vehicle. Different transportation modes are associated with substantially different service radii, route-selection mechanisms, and sensitivities to traffic congestion and pedestrian infrastructure. Consequently, an area that appears well covered under a motor-vehicle-based assessment may, in practice, constitute a service gap under walking or running modes due to limited pedestrian access or inadequate crossing facilities.

This study addresses two interrelated questions. First, to what extent do travel-mode assumptions change AED coverage estimates and the spatial configuration of optimal deployment? Existing AED location studies typically evaluate coverage under a single mode, yet lay responders may walk, cycle, or drive, and each mode implies a different service radius and route logic. Second, how can a risk-informed demand surface—derived from large-scale empirical OHCA records rather than generic population proxies—improve the realism of coverage optimization? Using 17,842 ambulance dispatch records from Wuhan, we construct an age-stratified potential OHCA demand surface and integrate it with multimodal transportation networks into LSCP and MCLP models under a 4-min threshold. We further compare baseline and optimized accessibility across five mobility scenarios to identify deployment priorities that are robust to mode heterogeneity.

## 2 Literature review

Spatial optimization models are a class of analytical and mathematical approaches used to identify optimal spatial arrangements of facilities, services, or resources across geographic space, and are particularly relevant for planning time-critical medical devices. Their theoretical foundations are well-established in operations research and geography (23). Classical spatial optimization models can be broadly categorized into two types: coverage-based and accessibility-based approaches. The former models originate from the LSCP, which seeks to cover all demand points using the minimum number of facilities within a predefined service threshold (11). A more pragmatic variant, the MCLP, aims to maximize the population or demand covered within a specified service radius—such as a time-based catchment—given a fixed number of facilities, thereby closely aligning with response-time-oriented planning standards (12). Extensions of coverage models have incorporated concepts like expected coverage (24), multiple coverage (25), and variations in facility types (26). Accessibility-based models, rooted in Hakimi's seminal work, include the p-median problem, which minimizes the total weighted travel distance or time between users and their nearest facility, with the objective of improving overall system efficiency (13).

Spatial optimization models are also widely implemented in AED deployment planning. Early AED placement was predominantly guided by coverage models like the MCLP. Recent research, however, reflects a trend toward more sophisticated, integrated, and context-aware frameworks. Methodological innovations in adjacent domains, such as stochastic demand modeling (27), dynamic dispatch optimization (28), and robust optimization approaches for medical service design (29), have also informed the evolution of AED location models. Tierney et al. (14) bridged risk prediction and location optimization for AEDs by combining Bayesian spatial models with exact allocation algorithms. Similar coverage-based approaches have been applied to other urban emergency services, such as multi-hazard emergency evacuation shelter siting (30). In high-density urban environments, Zhong and Wei (31) developed a multi-criteria framework optimized with machine learning and genetic algorithms, revealing facility density as more critical than population density in deployment strategy. Similarly, Chen et al. (16) implemented a comprehensive risk assessment using hexagonal grids to guide phased AED installation in Hangzhou, while Shi and Zhang (19) demonstrated through location-allocation modeling that strategic placement of a limited number of additional AEDs could achieve near-total coverage in an urban district. Some studies have focused on the geographic relationship between OHCA locations and the distances that community first responders need to travel to retrieve AEDs (32).

Parallel to model sophistication, the conceptualization of “accessibility” in EMS/AED research is expanding. Studies now emphasize the complete emergency care journey, advocating for models that account for both the scene response time and the subsequent transport time to hospital (33, 34). Crucially, research has identified that in vertical urban environments, the Patient Access Trip (PAT)—the time to reach a patient within a building—can be a significant and often overlooked component of total response delay, introducing new spatial equity challenges (34, 35). In parallel, AED-equipped drones have emerged as a complementary delivery strategy: real-world and experimental studies have shown that drones can shorten AED arrival or treatment-free intervals in selected emergency settings (36, 37). Furthermore, the imperative for equitable service distribution is driving methodological innovation. A growing body of work explicitly integrates equality objectives into location models, employing metrics such as the Gini coefficient (38), minimizing variance (39), or specifically targeting the reduction of coverage gaps between urban and rural areas (40).

Recent emergency-resource location-allocation research further distinguishes spatial equality from spatial equity. Song et al. (41) show that comparable spatial access across locations is conceptually distinct from allocation that explicitly accounts for heterogeneous vulnerability or need. In this sense, threshold-based covering models such as the LSCP primarily address spatial coverage equality, whereas stronger equity formulations require population-specific vulnerability-, need-, or disadvantage-sensitive objectives.

## 3 Study area and data sources

### 3.1 Study area

This study focuses on Wuhan, the capital city of Hubei Province, situated in the middle reaches of the Yangtze River. As the largest metropolis in central China and one of the country's nine National Central Cities, Wuhan plays a pivotal role in regional politics, economy, and culture. It covers a total area of 8,569.2 km^2^, administratively divided into 13 districts (seven central urban districts and six suburban districts). Demographic data from the Wuhan Statistical Bureau reveals key characteristics of its population. In 2024, the city's permanent resident population reached 13.81 million, showing a slight increase of 3.51 thousand from the previous year, primarily attributed to inter-city and rural-to-urban migration (42). The urbanization rate stands at 85.0%. Furthermore, the city is experiencing significant population aging. According to the latest census data, approximately 17.2% of the population was aged 60 and above, and 11.8% was aged 65 and above in 2020, representing increases of 4.5 and 3.7 percentage points, respectively, since 2010 (43). Since most OHCA events occur in the central urban district, the empirical study therefore designated Wuhan's main urban districts as the study area, with approximately 6.54 million residents.

### 3.2 Data sources and processing

The data utilized in this study encompassed population data, historical OHCA incident records, locations of existing AEDs, candidate sites for new AEDs, and geospatial transport networks. All spatial analyses were conducted using a consistent 1 km × 1 km grid as the fundamental unit for representing demand and calculating accessibility. Population data were obtained from the WorldPop gridded dataset (https://www.worldpop.org/), which provides high-resolution population estimates at a 100 m × 100 m resolution. For this study, the 2020 data were aggregated to the 1 km × 1 km analysis grid. To align with risk patterns, the population within each grid was stratified into four age groups (0–19, 20–39, 40–59, and ≥60 years) based on the demographic structure from local statistical yearbooks.

Historical Out-of-Hospital Cardiac Arrest incident data were provided by the Wuhan Emergency Medical Center, comprising 17,842 valid ambulance dispatch records for the years 2024–2025. The dataset includes key attributes such as anonymized incident location (aggregated to the community level) and patient age. These records were used to calculate age-stratified annual OHCA incidence rates, which inform the construction of a risk-weighted demand surface.

This study uses retrospectively archived, anonymized and aggregated OHCA records provided by the Wuhan Emergency Medical Center. All personal identifiers were permanently removed prior to data transfer. No living human participants were recruited, and only secondary spatial modeling analysis was conducted.

The label “2024–2025” indicates calendar years spanned by the OHCA dataset rather than a full 24-month observation window. The actual observation duration for valid OHCA records is approximately 1.41 calendar years. Person-years at risk in Table 1 were computed by multiplying gridded age–stratified resident population estimates by this exact observation duration. We use static 2020 WorldPop gridded population as the denominator, assuming minor net population change within this short study window. The aggregate 6.54 million represents official statistical resident population of main urban districts, which slightly differs from aggregated gridded WorldPop population used for person-year computation.

**Table 1:** Parameters for calculating the potential OHCA demand in Wuhan.

Age group (years)	Person-years at risk	Observed cases	Annual incidence rate
0–19	2,789,609.4	553	*r*_1_ = 0.0002
20–39	2,728,927.8	1,234	*r*_2_ = 0.00045
40–59	2,719,957.02	3,630	*r*_3_ = 0.0013
≥60	981,340.2	12,425	*r*_4_ = 0.013

The locations of 3,569 existing public-access AEDs in the main urban area were supplied by the Wuhan Emergency Medical Center. For potential new AED deployment, the centroids of grid cells were adopted as candidate sites. This grid-based candidate set ensures spatial exhaustiveness—every demand location is itself eligible to host a new AED—thereby avoiding arbitrary pre-selection bias and ensuring that the solutions are not artificially constrained by a limited pre-selected candidate pool. The 3,569 existing AEDs were treated as fixed (required) facilities that remain in service and contribute to baseline coverage; only the grid centroids were candidate locations for new deployment.

A detailed multimodal transportation network dataset was acquired from the Wuhan Geomatics Institute to model realistic travel paths for first responders. Separate network layers were constructed for three primary modes: driving, cycling, and walking. Travel speeds were assigned to network segments based on road hierarchy and mode characteristics, as detailed in Table 2. The study area, ring-road structure, population-density background, water bodies, and existing AED distribution are summarized in Figure 1. Major rivers and lakes were treated as physical barriers in the network analysis, with cross-water movement permitted only through mapped roads, bridges, or other valid network connections; thus, water bodies can increase access time by forcing detours to available crossings. The spatial configuration of the multimodal transportation network, including expressways, major and minor arterials, residential roads, and dedicated cycling routes, is illustrated in Figure 2.

**Table 2:** Assigned travel speeds for multimodal network analysis.

Road type	Road subtype	Assigned speed (km/h)
Expressway	Urban expressways and ring roads with high-speed design standards	100
Major arterial	Primary trunk roads and main urban thoroughfares	80
Minor arterial	Secondary arterial roads and collector streets with frequent access	40
Residential	Local streets, neighborhood roads, and commercial area internal roads	30
Cycling	Dedicated bicycle lanes and roads where cycling is permitted	15
Walk	Sidewalks, pedestrian pathways, and public areas	5

Road classification is based on the transportation network dataset obtained from the Wuhan Geomatics Institute. Speed assignments reflect common design speeds and regulatory limits according to Chinese urban road standards.

**Figure 1:**
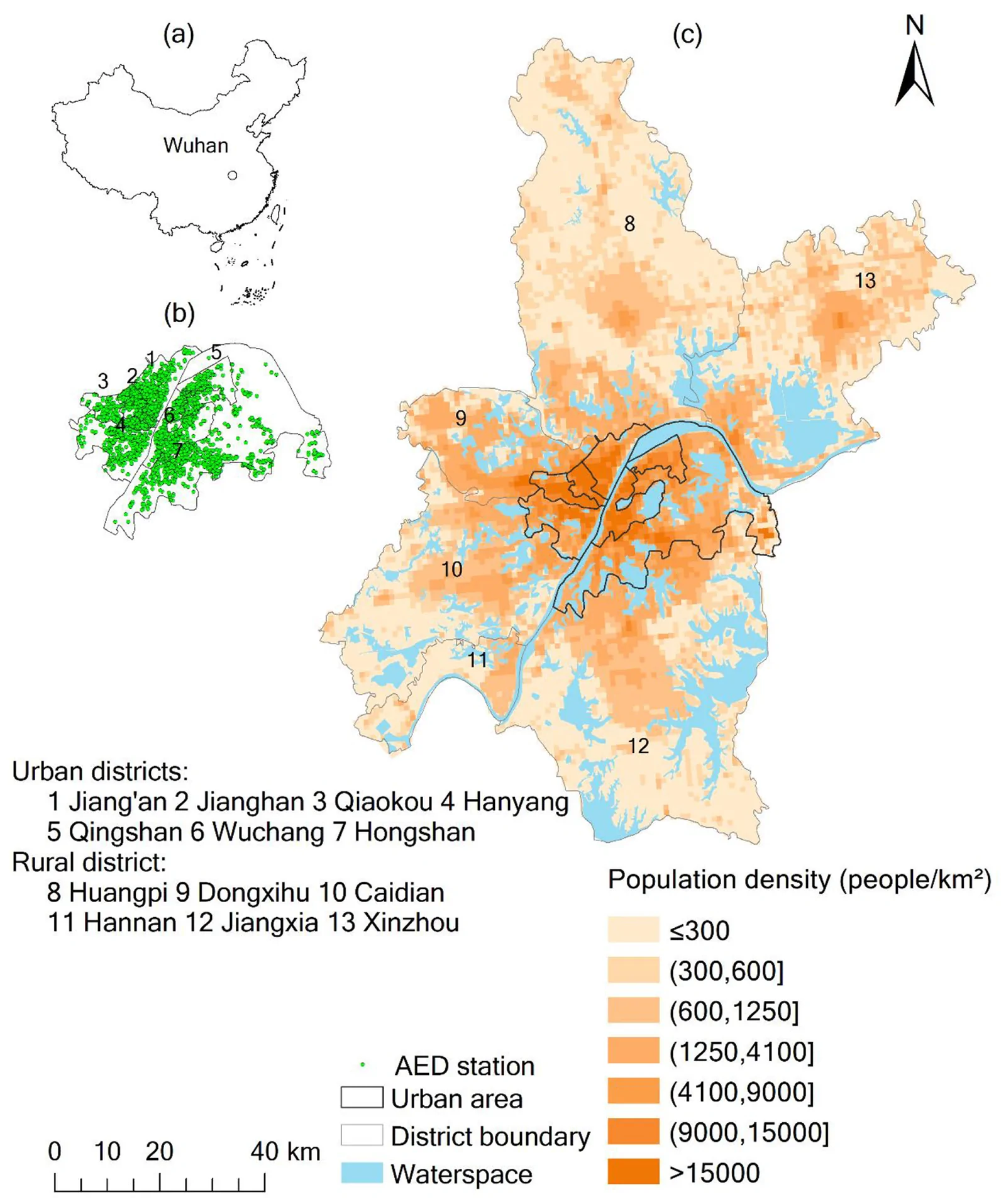
Study area. **(a)** Wuhan within China; **(b)** Wuhan within Hubei; **(c)** Wuhan study area.

**Figure 2:**
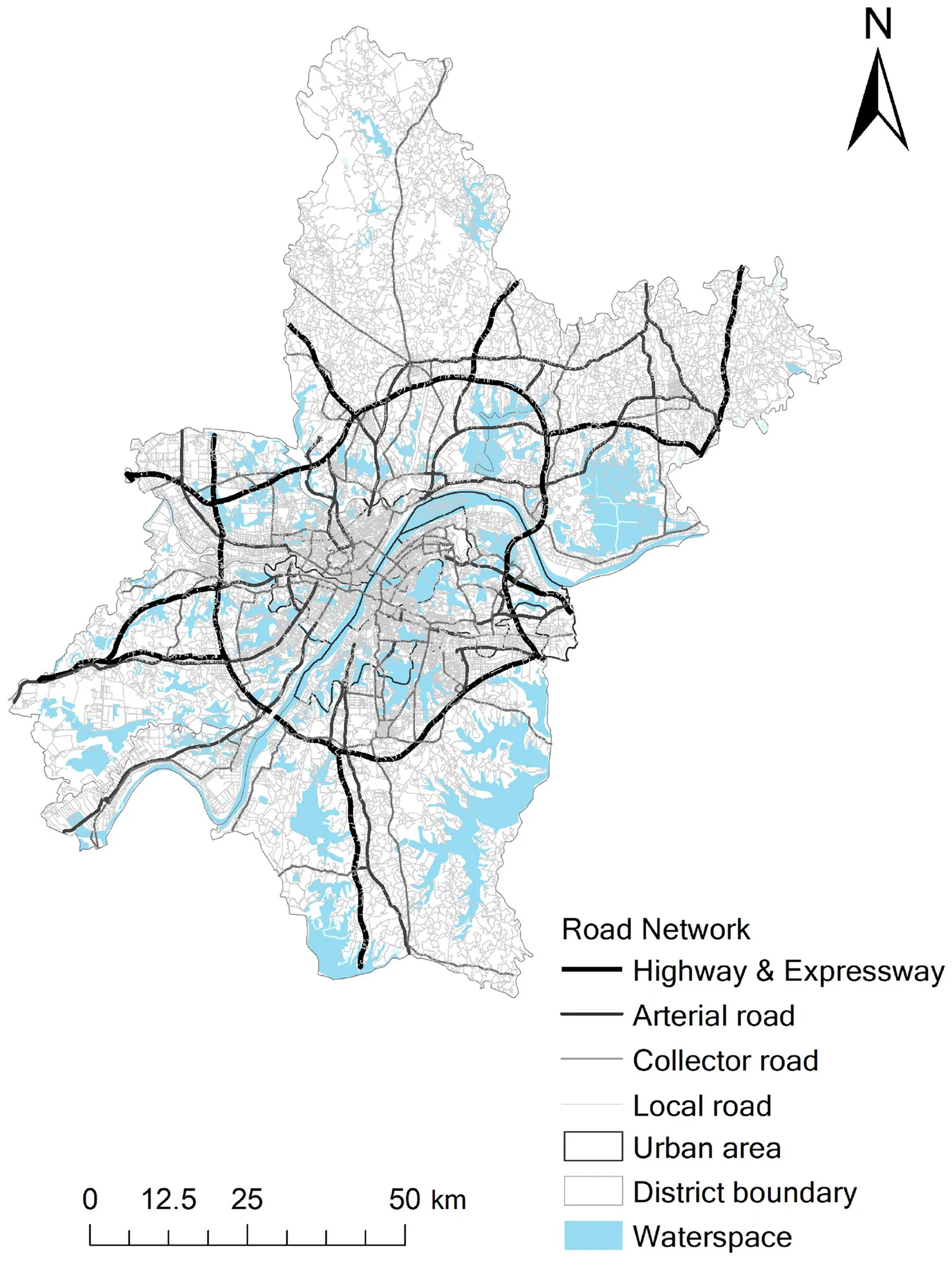
Multimodal transportation network of Wuhan.

All spatial datasets were projected to a unified coordinate system (CGCS2000). The centroids of the 1,161 populated grid cells within the study area were defined as demand points (*i* ε I). A forward-looking “Potential OHCA Demand” for each grid was calculated by applying the derived age-specific incidence rates to the corresponding resident population, generating a continuous, non-zero demand surface for optimization.

### 3.3 Calculation of potential OHCA demand

To construct a non-zero risk-weighted demand surface for optimization, we developed a “Potential OHCA Demand” metric based on age-specific OHCA incidence and population exposure. Historical OHCA records were used to estimate city-wide age-specific annual incidence rates. These rates were then applied to the age-stratified population in each grid. Importantly, the geographic coordinates of the 17,842 historical incidents do not enter Equation 1 directly; therefore, the resulting surface should be interpreted as an age-risk-weighted population demand surface rather than a smoothed map of historical incident locations.

The potential demand *Di* for grid cell *i* is given by:


$$\begin{array}{l} D_{i}=\overset{n}{\underset{a=1}{\sum }}\left(r_{a}\times P_{a,\;i}\right) \end{array}$$ (1)

where *r*_*a*_ is the annual incidence rate for age group *a*, and *P*_*a, i*_ is the corresponding population in grid *i*. This approach ensures that areas with a higher proportion of vulnerable age groups, particularly older adults, are appropriately weighted, generating a continuous risk-weighted demand surface without zero values.

This demand surface serves as the primary input for all subsequent location-allocation models, ensuring that optimization targets areas of anticipated risk rather than solely historical incident locations.

### 3.4 Methodology for multimodal AED optimization

Traditional AED location models typically optimize coverage based on a single transportation mode, most commonly vehicular travel (12). However, this approach fails to capture the multimodal reality of urban emergency response, where lay responders may utilize walking, cycling, or driving depending on context and accessibility. To address this limitation, this study extends two classical facility location models—the LSCP and the MCLP—by incorporating weighted multimodal transportation networks. In this study, access time is defined as the one-way network travel time from an AED to the demand-grid centroid (representing the OHCA location)—that is, the time a lay responder would take to retrieve the AED and reach the patient. We adopt a 4-min threshold for this one-way trip, consistent with prior work using 3–4-min walking catchments (44) and with Khan et al. (45).

The core innovation lies in defining a weighted generalized travel time under each mobility scenario. Rather than assuming that weighted multimodal accessibility is achieved by whichever mode is fastest, this study represents weighted multimodal accessibility as a weighted combination of mode-specific travel times. In the weighted multimodal scenarios, the travel time is assumed to be jointly composed of two transport modes. This setting provides a transparent and parsimonious representation of multimodal accessibility in the absence of individual-level emergency trajectory data. It should be noted that the weighted generalized travel time is not intended to represent a single realizable trip by an individual responder. Rather, it operationalizes a planning-level assumption about the modal composition of the responder population: θ represents the assumed share of responders relying on a given mode, so that the weighted time reflects an expected access time across heterogeneous responders. In this sense, the Drive + Walk and Cycling + Walk scenarios should be interpreted as weighted multimodal accessibility scenarios, not as literal sequential trip times. The analysis was conducted with the following notation:

*i, j*: indices of demand points and candidate AED locations, respectively;

*I, J*: sets of demand points and candidate AED locations, respectively; where both are defined on the same 1,161 grid-cell centroids ((|*I*| = |*J*| = 1,161));

*F*: set of existing (fixed) AED locations, with (|*F*| = 3,569);

*A*: set of transportation modes, where *A* = {*d, b, w*}, representing driving, cycling, and walking, respectively;

*D*_*i*_: potential OHCA demand at demand point *i* (calculated via Equation 1 in Section 3.3);

$\begin{array}{c} t_{ij}^{a} \end{array}$: shortest-path travel time from candidate AED location *j* to demand point *i* using transport mode *a*;

θ_*a*_: population share of lay responders adopting travel mode a within the planning scenario, satisfying 0 ≤ θ_*a*_ ≤ 1 and $\underset{a\in A}{\sum }\theta_{a}=1$. This weight represents the proportional composition of responders across mobility groups, not the fraction of total travel time;

*t*_*ij*_: weighted expected multimodal travel time from demand point i to candidate AED location *j* in a weighted multimodal scenario, defined as


$$\begin{array}{l} t_{ij}=\underset{a\in A}{\sum }\theta_{a}t_{ij}^{a} \end{array}$$ (2)

T_*c*_: Critical 4-min response time threshold;

*p*: Number of new AEDs to be sited;

*N*_*i*_: set of candidate AED locations that can cover demand point i within the critical response time threshold, defined as Equation 3

*a*_*if*_: Binary parameter; *a*_*if*_ = 1 if pre-existing AED f in F covers demand point i within the critical response-time threshold T_*c*_, and *a*_*if*_ = 0 otherwise.


$$\begin{array}{l} N_{i}=\left\{j\varepsilon J|t_{ij}\leq T_{c}\right\} \end{array}$$ (3)

The weighted expected travel time formulation in Equation 2 reflects a planning-level expectation for heterogeneous responder subgroups. For each demand point *i*, we first calculate multimodal travel time to every candidate facility *j* and then select the site with minimal *t*_*ij*_. This simplification assumes all responders at *i* use the same AED facility, regardless of their travel mode. A stricter alternative would find the nearest facility independently for each mode and average those minimal travel times, allowing different-mode responders to access distinct sites. We adopt our current approach for computational feasibility in this large-scale urban model and note it as a modeling simplification. Notably, coverage based on population-mean expected travel time can label a demand point as covered even if slower-mode subgroups exceed the 4-min cutoff. Our *N*_*i*_ rule implements mean-based coverage rather than subgroup-specific guarantees. Separate time constraints for each mobility group would require extended multi-constraint models and is left for future research.

Decision variables:


$$X_{j}=\{\begin{array}{cc} 1 & \text{if an AED is placed at candidate location j}\\ 0 & \text{otherwise} \end{array}$$ (4)

$$Y_{i}=\{\begin{array}{cc} 1 & \text{if demand point i is covered within the critical}\\ \; & \text{response time threshold}\\ 0 & \text{otherwise} \end{array}$$ (5)

#### 3.4.1 Multimodal location set covering problem (M-LSCP)

Based on the weighted generalized travel time defined above, the M-LSCP determines the minimum number of AEDs required to achieve universal coverage of all demand points:


$$\begin{array}{l} \text{Minimize }Z_{1}=\underset{j\in J}{\sum }X_{j} \end{array}$$ (6)

subject to:


$$\begin{array}{l} \underset{j\in J}{\sum }a_{ij}x_{j}+\underset{f\in F}{\sum }a_{if}\geq 1,\;\forall i\in I \end{array}$$ (7)

$$\begin{array}{l} X_{j}\in \left\{0,1\right\},\;\forall j\in J \end{array}$$ (8)

#### 3.4.2 Multimodal maximal covering location problem (M-MCLP)

In the M-MCLP formulation, pre-existing fixed AED facilities from set *F* are always active and contribute to demand-point coverage alongside newly-selected candidate sites *j* in *J*.

Given practical budget constraints, the M-MCLP identifies priority locations that maximize covered demand based on the weighted generalized travel time structure shown in Equations 9–13:


$$\begin{array}{l} \text{Maximize }Z_{2}=\underset{i\in I}{\sum }D_{i}\cdot Y_{i} \end{array}$$ (9)

subject to:


$$\begin{array}{l} \underset{j\in N_{i}}{\sum }X_{j}+\underset{f\in F}{\sum }a_{if}\geq Y_{i},\;\forall i\in I \end{array}$$ (10)

$$\begin{array}{l} \underset{j\in J}{\sum }X_{j}=p \end{array}$$ (11)

$$\begin{array}{l} X_{j}\in \left\{0,1\right\},\;\forall j\in J \end{array}$$ (12)

$$\begin{array}{l} Y_{i}\in \left\{0,1\right\},\;\forall i\in I \end{array}$$ (13)

#### 3.4.3 Solution approach

Both optimization models were solved using the heuristic Location-Allocation solver in ArcGIS Network Analyst. While this solver does not guarantee global optimality for large instances, its solutions are widely used as robust planning references in GIS-based facility siting studies (16, 19). We therefore interpret our results as planning benchmarks rather than provably optimal configurations. Three separate network datasets were created: pedestrian (incorporating sidewalks and crosswalks), cycling (including dedicated bike lanes and shared-bike station access), and vehicular (based on the complete road hierarchy). Travel times were calculated using mode-specific speeds: walking (5 km/h), cycling (15 km/h), and driving (adjusted for road type). ArcGIS is used here as an analyst-facing planning and decision-support environment for public-health and urban-planning agencies; it is not assumed to be software used by bystanders during an actual emergency.

To systematically examine the impact of transportation assumptions, five mobility scenarios were analyzed:

(i) Drive-only: accessibility based solely on the vehicular network;
(ii) Cycling-only: accessibility based solely on the cycling network;
(iii) Walk-only: accessibility based solely on the pedestrian network;
(iv) Drive + Walk: weighted multimodal accessibility represented by a weighted combination of driving and walking travel times, with each mode assigned a 50% population-share weight among lay responders;
(v) Cycling + Walk: weighted multimodal accessibility represented by a weighted combination of cycling and walking travel times, with each mode assigned a 50% population-share weight among lay responders.

The equal-weight setting does not imply that the two modes contribute equally in every real-world emergency trip; rather, it serves as a parsimonious scenario-based approximation for multimodal accessibility analysis.

#### 3.4.4 Performance evaluation metrics

To comprehensively assess optimization outcomes, four metrics were calculated:

Demand-weighted coverage rate (DCR): Percentage of modeled potential OHCA demand covered within 4 min, as shown in Equation 14


$$\begin{array}{l} DCR=\frac{\sum_{i:Y_{i}=1}D_{i}\cdot y_{i}}{\sum_{i\in I}D_{i}}\times 100\% \end{array}$$ (14)

Geographic coverage rate (GCR): Percentage of grid cells with at least one accessible AED, as shown in Equation 15


$$\begin{array}{l} GCR=\frac{\left|\left\{i:Y_{i}=1\right\}\right|}{\left|I\right|}\times 100\% \end{array}$$ (15)

Mean shortest access time (MSAT): Demand-weighted average travel time to the nearest AED, as shown in Equation 16


$$MSAT=\frac{\sum_{i\in I}D_{i}\cdot \min\left(\underset{j\in J:X_{j}=1}{\min}t_{ij},\underset{f\in F}{\min}t_{if}\right)}{\sum_{i\in I}D_{i}}$$ (16)

The minimum travel time for each demand point *i* is evaluated across both newly-selected candidate AED locations (*j* in *J*, *X*_*j*_=1) and all pre-existing fixed AED locations (*f* in *F*).

MSAT-Std: Demand-weighted standard deviation of shortest access time, used to describe the dispersion of access times across demand locations, as shown in Equation 17.


$$\begin{array}{l} MSAT-Std=\\ \sqrt{\frac{\sum_{i\in I}D_{i}\;\cdot \;{\left(\min\left(\underset{j\in J:X_{j}=1}{\min}t_{ij},\underset{f\in F}{\min}t_{if}\right)-MSAT\right)}^{2}}{\sum_{i\in I}D_{i}}} \end{array}$$ (17)

## 4 Results

### 4.1 Modeled potential OHCA demand surface

To establish a risk-informed foundation for AED deployment, we analyzed 17,842 OHCA incidents recorded in Wuhan's main urban area from 2024 to 2025. These incidents were spatially aggregated into 1,161 one-square-kilometer grid cells for analysis.

This “Potential OHCA Demand” (Figure 3) combines two components: (1) age-specific OHCA incidence rates estimated from historical records, and (2) age-stratified population exposure in each grid. The integration of these components ensures that the resulting demand surface has no zero-value grids, thereby providing a realistic and comprehensive representation of where AEDs are needed to serve the population at risk, not merely where incidents have recently occurred.

**Figure 3:**
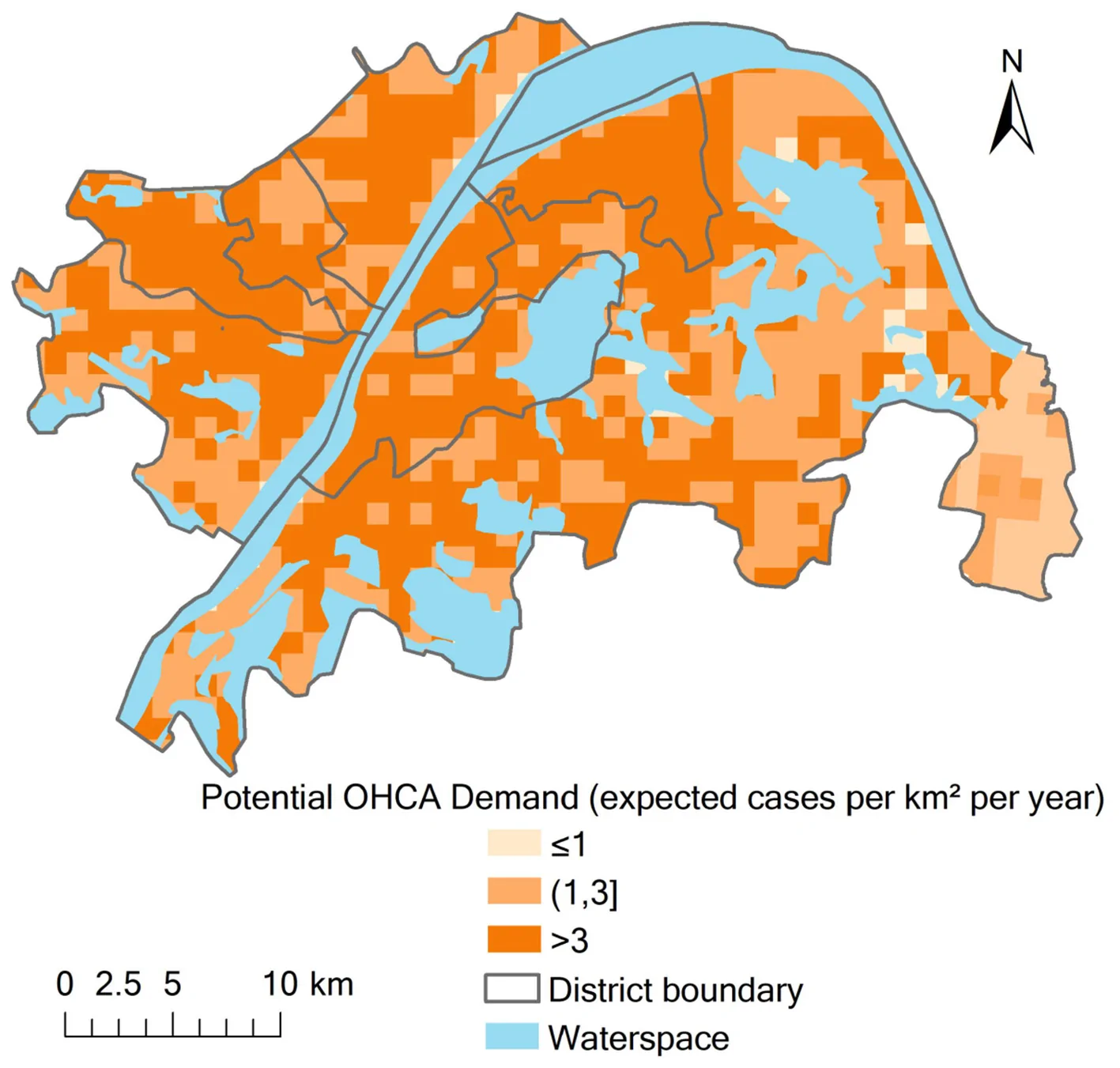
Spatial distribution of modeled potential OHCA demand.

Risk stratification of this optimized demand surface validates its rationale and establishes priority zones. Figure 3 presents boxplots comparing the “Potential OHCA Demand” across the three urban rings. The analysis confirms a persistent core-periphery gradient but with a moderated disparity. Critically, the median demand outside the Third Ring is now substantively greater than zero, correctly reflecting the underlying population risk in these areas rather than the statistical noise of short-term incidence. Furthermore, Figure 4 shows that areas within the Second Ring Road exhibit a wider interquartile range and several high-value outliers, indicating substantial intra-zone heterogeneity in potential OHCA demand. This suggests that, despite the generally higher demand within the urban core, the burden is unevenly distributed, with localized hotspots accounting for disproportionately high levels of potential demand.

**Figure 4:**
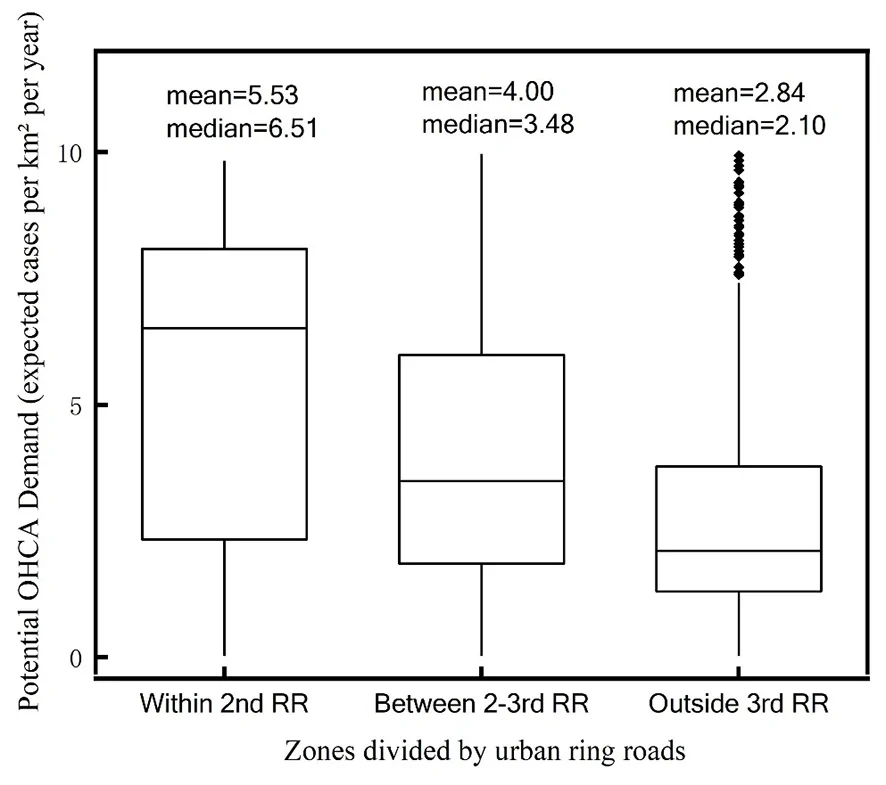
Comparison of potential OHCA demand across urban ring zones. RR, ring road.

Therefore, the “Potential OHCA Demand” surface (Figure 3) serves as the spatially continuous demand input for all subsequent location-allocation optimization. The high-demand grids within the Second Ring, particularly the outlier hotspots, are designated as Priority 1 demand zones. The secondary elevated-demand zones in western Hongshan and southern Qingshan are designated as Priority 2 demand zones. This age-risk-weighted demand scheme allows our LSCP and MCLP models (Sections 3.4.1, 3.4.2) to optimize AED placement according to the modeled distribution of population-weighted OHCA risk rather than relying solely on recent incident locations.

### 4.2 Baseline accessibility assessment via shortest-path network analysis

To quantify the performance deficiencies of the existing AED network and establish a benchmark for optimization, we conducted a city-wide shortest-path network analysis. For each of the 1,161 demand grids, the travel time to the nearest existing AED was calculated under five transportation modes: Driving, Cycling, Walking, Drive + Walk, and Cycling + Walk, using the actual urban road network and assigned mode-specific speeds.

Baseline accessibility varied markedly across travel modes, as shown in Figures 5a–e. Driving provided the most extensive coverage within the 4-min threshold, followed by cycling, whereas walking produced the most fragmented and limited accessibility pattern. The weighted Drive + Walk and Cycling + Walk scenarios showed intermediate spatial patterns between the faster modes and walking. Areas with travel times exceeding 8 min were concentrated primarily in peripheral and poorly connected locations, although localized gaps were also evident within the urban core. These spatial differences indicate that estimated AED accessibility is highly sensitive to travel-mode assumptions, and assessments based solely on driving may overestimate practical accessibility for non-driving responders.

**Figure 5:**
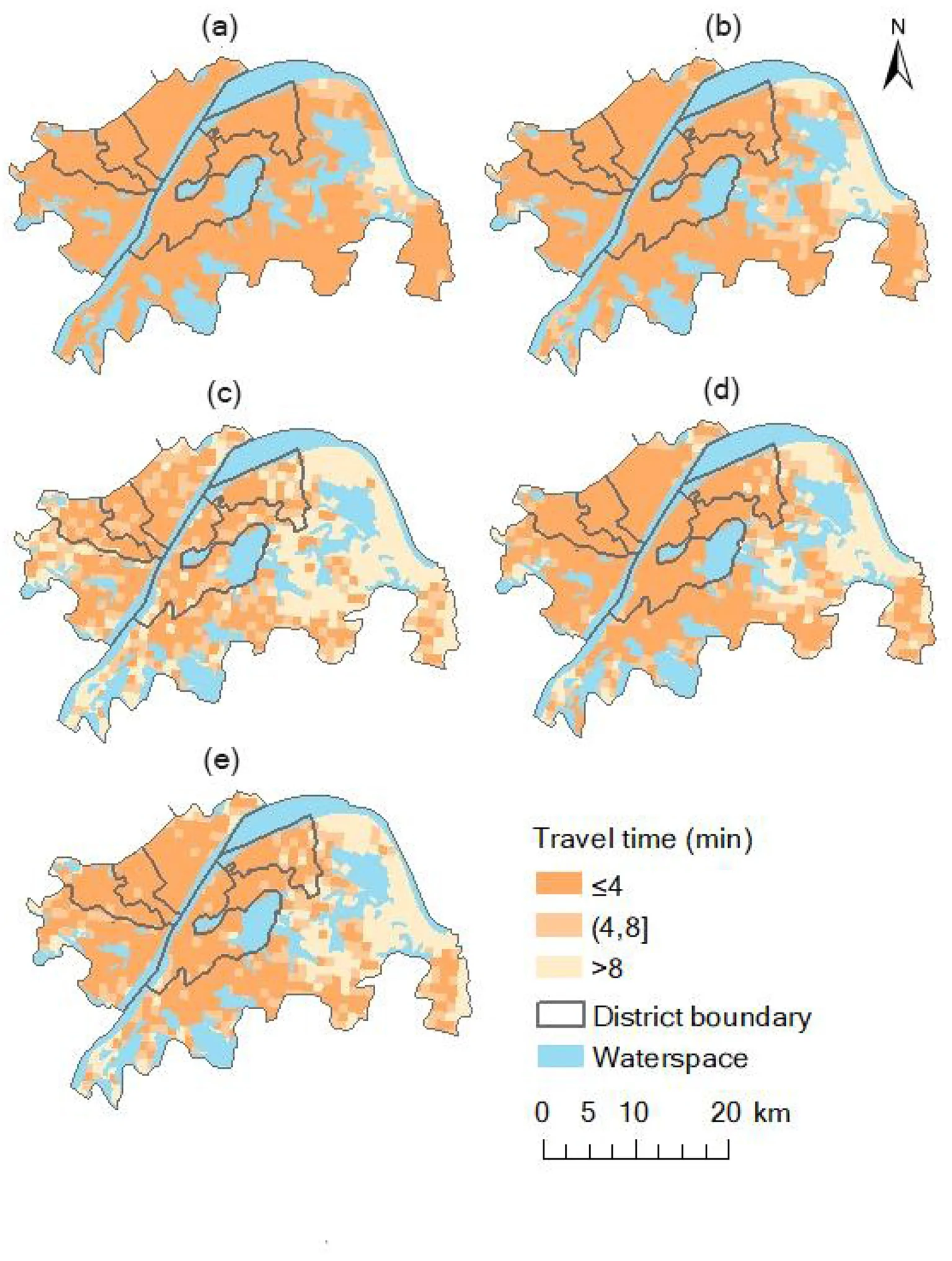
Baseline one-way accessibility time from each demand-grid centroid to the nearest existing AED: **(a)** driving; **(b)** cycling; **(c)** walking; **(d)** drive + walk weighted accessibility; **(e)** cycling + walk weighted accessibility.

### 4.3 A multi-modal LSCP analysis

A quantitative summary across all five modes uncovers substantial accessibility disparities. The baseline performance metrics are detailed in Table 3. Two key patterns emerge: (1) Accessibility is strongly dependent on transportation mode, with demand-weighted coverage ranging from 85.07% under Driving to 31.55% under Walking; and (2) Both demand-weighted and geographic coverage are substantially lower under walking and cycling scenarios than under driving. Under the Drive + Walk scenario, DCR and GCR are 53.65% and 55.54%, respectively, within the 4-min service threshold. These results suggest that assessments based primarily on vehicular accessibility may overestimate the practical accessibility of the existing AED network for populations relying on walking or cycling.

**Table 3:** Baseline performance of the existing AED network within the 4-min response threshold.

Transportation mode	DCR (%)	GCR (%)	Interpretation
Driving	85.07	83.76	High demand-weighted coverage; some geographic gaps remain
Cycling	68.27	68.51	Moderate coverage under the cycling network
Walking	31.55	33.94	Limited accessibility under the walking assumption
Drive + walk	53.65	55.54	Intermediate coverage under weighted driving and walking access
Cycling + walk	45.41	47.28	Intermediate coverage under weighted cycling and walking access

DCR, demand-weighted coverage rate; the percentage of modeled potential OHCA demand covered within the 4-min critical response threshold; GCR, geographic coverage rate; the percentage of 1 km × 1 km grid demand points covered within the 4-min threshold.

This spatial and quantitative diagnosis establishes a clear imperative for optimization. The identified gaps—both in the expansive suburban periphery and within high-density urban cores—directly conflict with the spatial distribution of cardiac arrest risk (“Potential OHCA Demand”, Figure 3). The severe underservice quantified in Table 3, particularly for non-driving populations, provides strong evidence that strategic, multimodal AED deployment can improve coverage in underserved areas and support a more balanced public health response system.

LSCP model was employed to determine the minimum number of new AEDs required to achieve 100% coverage of all demand points within the critical 4-min response window, under five distinct transportation mode scenarios. The spatial configurations of these full-coverage blueprints are presented in Figure 6, and the associated resource requirements and accessibility impacts are quantified in Table 4.

**Figure 6:**
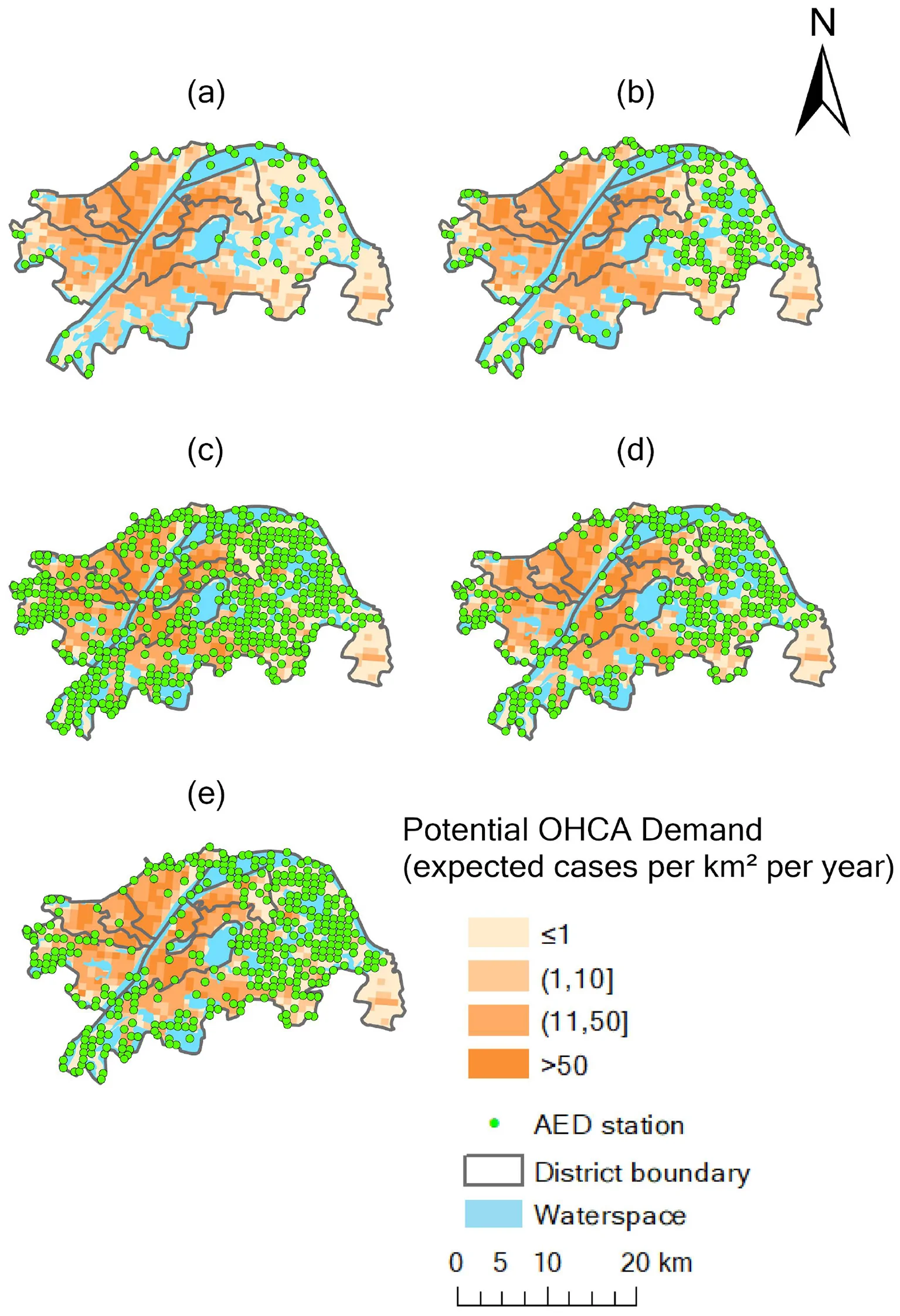
LSCP optimization results under different travel modes: **(a)** driving; **(b)** cycling; **(c)** walking; **(d)** drive + walk; **(e)** cycling + walk. Sites in lower-density areas can be selected when they close otherwise-uncovered network gaps within the threshold.

**Table 4:** System performance of the LSCP-derived universal coverage blueprints.

Transportation mode	MSAT-mean (pre)	MSAT-std (pre)	MSAT-mean (post)	MSAT-std (post)	AEDs required
Driving	2.53	3.39	1.26	1.06	68
Cycling	4.51	6.41	1.46	1.10	176
Walking	15.02	21.36	1.50	1.28	521
Drive + walk	7.26	8.88	1.64	1.18	327
Cycling + walk	9.76	13.88	1.65	1.22	381

The analysis first quantified the stark trade-off between spatial equity and resource investment. The number of AEDs needed for universal coverage varied by an order of magnitude depending on the assumed travel mode (Drive only: 68 vs. Walk only: 521). The weighted multimodal Drive + Walk mode presented a pragmatically balanced requirement (327 AEDs), illustrating the resource implications of moving from an exclusionary (driver-only) to an inclusive (multi-modal) coverage standard.

Distinct spatial deployment logics emerged under different mobility assumptions. The Drive-only LSCP blueprint (Figure 6a) adopted a “strategic-outpost” pattern, efficient for car-owners but leaving substantial spatial gaps for non-driving populations. The Walk-only blueprint (Figure 6c) forced a “saturation-infill” pattern in the urban core, which, while spatially comprehensive, was prohibitively resource-intensive. Crucially, the Drive + Walk blueprint (Figure 6d) exhibited a “balanced-hub” pattern, distributing sites along major corridors and transit nodes to improve coverage under the weighted multimodal scenario. Some selected sites occur in relatively low-density peripheral areas because LSCP prioritizes closing residual coverage gaps across the entire modeled demand set rather than ranking sites solely by local population density; network barriers, long detours, and sparse existing AED provision can therefore make lower-density locations necessary for threshold-based coverage.

Finally, The Drive + Walk LSCP solution reduced the demand-weighted mean shortest access time from 7.26 min to 1.64 min. However, Universal coverage under this weighted expected-time criterion guarantees that the population-mean travel time meets the threshold, but it does not ensure every mobility subgroup satisfies the 4-min requirement. Therefore, full threshold coverage does not necessarily imply equitable accessibility across population subgroups, because mode-specific access times may still vary substantially across demand locations. We therefore interpret the LSCP solution as a coverage-oriented planning benchmark rather than an equity-optimal configuration. This distinction is consistent with prior emergency-resource location-allocation research separating spatial equality from need-sensitive spatial equity (41).

### 4.4 A multi-modal MCLP analysis

Given the substantial resource demands for full LSCP-based universal coverage, phased implementation is practically essential. We therefore applied the M-MCLP, constraining the number of new AEDs to 20% of the corresponding LSCP requirement (rounded to the nearest integer) to identify optimal first-phase locations that maximize immediate demand-weighted coverage. The objective was to identify the optimal first-phase locations that maximize immediate demand-weighted coverage. The resulting priority locations are shown in Figure 7, and their performance is evaluated in Table 5.

**Figure 7:**
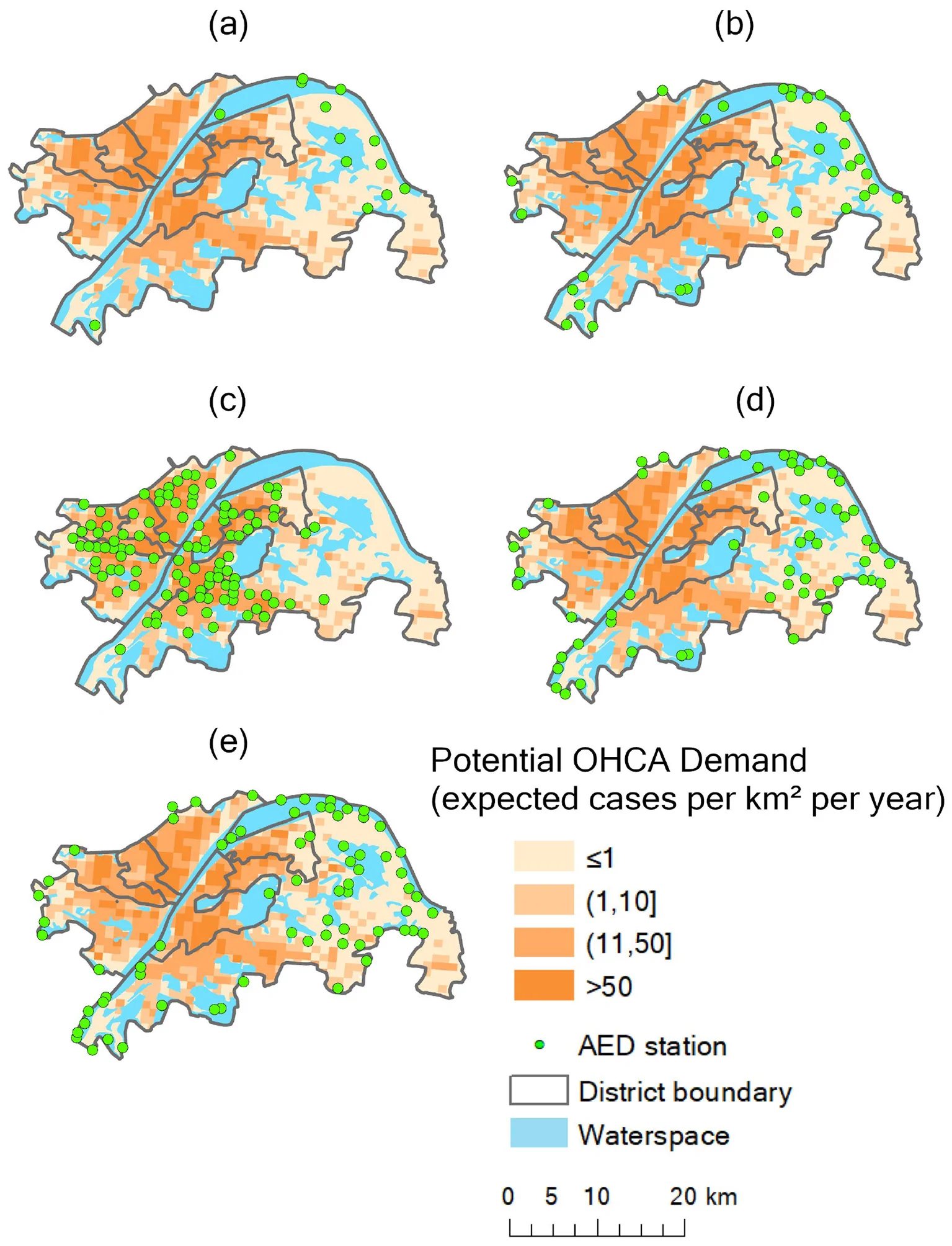
MCLP optimization results under different travel modes: **(a)** driving; **(b)** cycling; **(c)** walking; **(d)** drive + walk; **(e)** cycling + walk. Lower-density sites can be selected when they provide large marginal coverage gains across previously underserved network areas.

**Table 5:** Performance evaluation of the first-phase MCLP priority deployment.

Transportation mode	MSAT-mean (pre)	MSAT-std (pre)	MSAT-mean (post)	MSAT-std (post)	AEDs required	DCR (post)	GCR (post)
Driving	2.53	3.39	2.21	2.86	14	93.56	92.32
Cycling	4.51	6.41	3.84	5.11	35	82.87	83.28
Walking	15.01	21.36	10.08	15.23	104	45.55	48.57
Drive + walk	7.26	8.89	4.57	6.38	65	73.31	74.73
Cycling + walk	9.76	13.88	6.50	9.72	76	71.53	69.86

The MCLP-derived priority sites were not merely random subsets of their LSCP counterparts but represented the highest-coverage-yield locations within each spatial logic. For instance, the 65 sites selected in the Drive + Walk scenario (20% of 327 ≈ 65) yielded large marginal coverage gains across previously underserved demand grids (Figure 7d). *Post-hoc* visual inspection suggests that some selected sites are located near major transport corridors or interchange areas; however, transit-stop and park-and-ride data were not included as optimization inputs. This observation should therefore be interpreted qualitatively rather than as a site characteristic identified by the optimization model.

As shown in Figure 8, the marginal coverage gain declines as the number of new AEDs increases: the DCR rises rapidly with the first facilities and gradually saturates as the deployment approaches the LSCP requirement. This diminishing-return pattern indicates that a modest first-phase investment captures most of the achievable coverage improvement.

**Figure 8:**
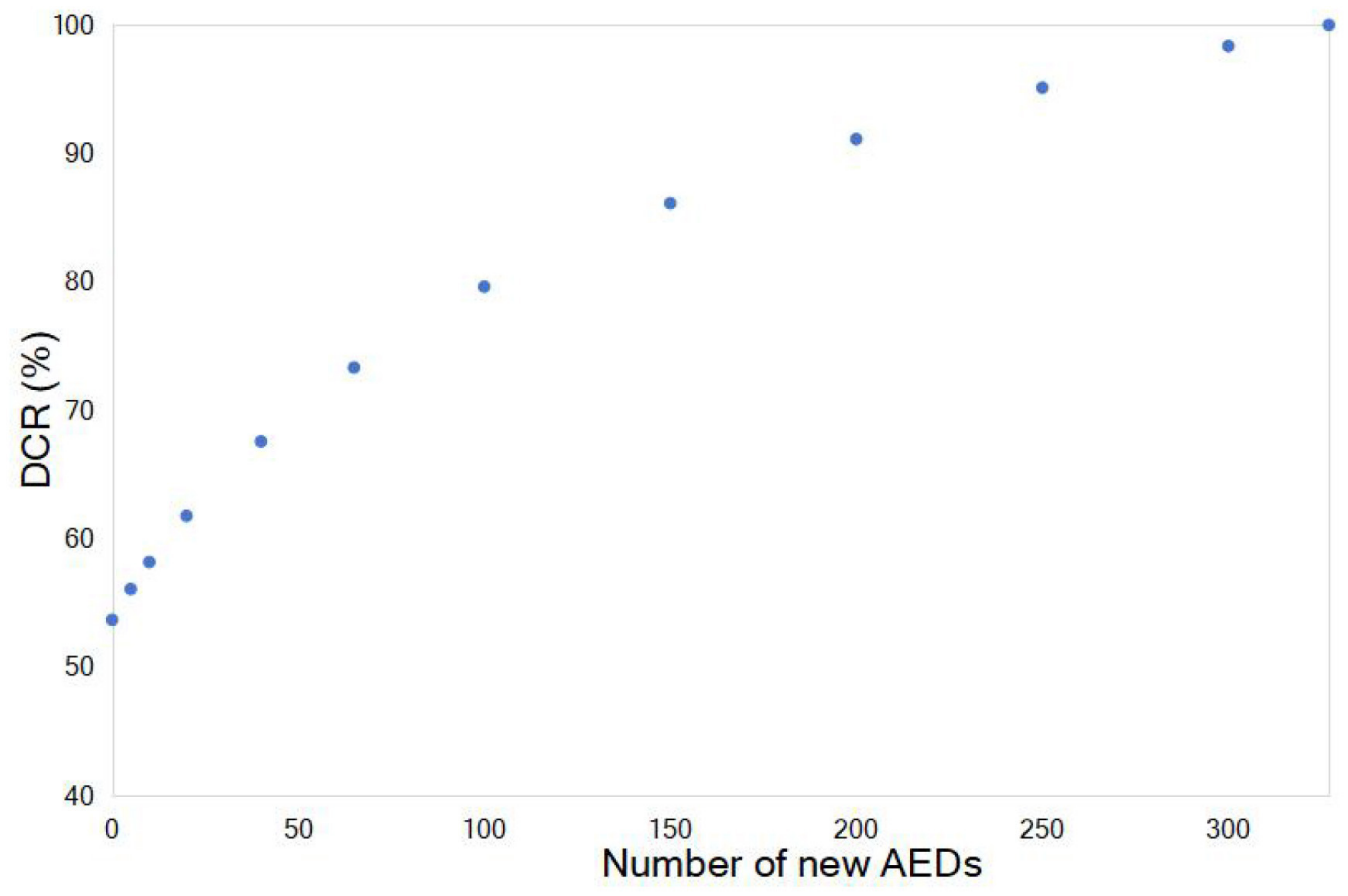
Diminishing marginal gain in demand-weighted coverage rate (DCR) with increasing numbers of newly-deployed AEDs.

The comparative analysis in Figures 7a–e demonstrates that travel-mode assumptions change both the estimated coverage pattern and the spatial locations prioritized by the MCLP. The driving mode exhibits extensive and continuous service coverage, aligning well with areas of high incidence, yet it may overestimate service capacity in core zones and obscure localized accessibility gaps under walking conditions. In contrast, the walking and cycling modes reveal highly fragmented, point-like distributions, showing substantial service gaps even in core zones with high modeled demand and thereby producing much lower estimated coverage under non-motorized access assumptions. The multi-mode composite scenarios (Figures 7d, e) yield coverage levels intermediate between the two, further highlighting the service limitations of individual AED sites under weighted multimodal assumptions. This comparison shows that single-mode assumptions can produce substantially different estimates of coverage and underserved areas, which may in turn affect subsequent resource-allocation decisions. Therefore, in AED network planning, integrating multimodal accessibility with modeled OHCA risk can support a more balanced, efficient, and spatially targeted emergency service system.

Budget constraints are ultimately determined by the unit cost of AED procurement, installation, and maintenance, which varies by site type. The 20% budget level adopted here reflects a pragmatic assumption that a first-phase investment remains financially feasible; it is not derived from a specific cost model. Combined with the marginal coverage analysis, unit-cost information would allow planners to identify the deployment scale at which the benefit-per-cost ratio begins to decline.

### 4.5 Sensitivity analysis of mode weights

Table 6 reports the number of AEDs required for universal coverage under the LSCP as θ varies from 0.3 to 0.7, together with the pure-mode endpoints (θ = 0 and θ = 1.0). To examine the sensitivity of the optimization results to the assumed mode weights in the weighted multimodal scenarios, we re-solved the M-LSCP model under alternative weight settings of θ = 0.3 to θ = 0.7, in addition to the baseline θ = 0.5, holding all other parameters constant.

**Table 6:** Sensitivity analysis of mode weights (θ) for the weighted multimodal scenarios.

θ	LSCP: AEDs required	LSCP: AEDs required
	Drive + walk	Cycle + walk
0	521	521
0.3	407	440
0.5	327	381
0.7	224	312
1.0	68	176

θ is the weight assigned to the faster mode (driving or cycling) in each weighted multimodal scenario, with 1 – θ assigned to walking. θ = 0 and 1.0 are single-mode endpoints (walking-only and driving/cycling-only, respectively).

Detailed numerical outcomes for different θ values are presented in Table 6 (see Section 4.5), together with the pure-mode endpoints (θ = 0 and θ = 1.0) for reference. The required number of AEDs decreased monotonically with increasing θ: for Drive + Walk, it declined from 407 at θ = 0.3 to 224 at θ = 0.7 (327 at θ = 0.5); for Cycling + Walk, it declined from 440 to 312 (381 at θ = 0.5). At every weight level, Drive + Walk required 15–33 fewer AEDs than Cycling + Walk, indicating that vehicle-assisted access is the more efficient supplementary mode. Even under the most conservative conservative multimodal weight (θ = 0.3), both weighted multimodal scenarios required substantially fewer AEDs than the walking-only baseline (521), confirming that multimodal configurations consistently reduce the resource burden of universal coverage.

Overall, the sensitivity analysis confirms that the qualitative findings—the ordering of the five scenarios, the advantage of multimodal configurations over single-mode walking, and the identification of priority deployment locations across weighted multimodal scenarios-are robust to the weight assumption. The absolute number of required AEDs varies with θ as expected, since a larger weight assigned to the faster mode (driving or cycling) expands the effective service radius of each facility; however, none of the central conclusions change across the tested range.

## 5 Discussion

This study systematically investigates how assumptions regarding transportation modes fundamentally shape AED network optimization. Two critical insights emerge. First, there is a marked discrepancy in demand-weighted coverage depending on the travel mode considered. The baseline assessment shows DCR values of 85.1% for driving, 53.7% for Drive + Walk, and 31.6% for walking. This demonstrates that network performance is not an absolute metric but is relative to the mobility options of the population. Second, and more consequentially, the transportation mode dictates the very logic of optimal resource allocation. The number of AEDs required for universal coverage varies by an order of magnitude across modes. Furthermore, the geographic priorities for deployment shift entirely—from a few strategic outposts suitable for drivers to a dense saturation of sites needed for pedestrians—even when aiming to cover the same number of people. This confirms that there is no single “optimal” network, but rather a spectrum of optimal configurations, each contingent on a specific vision of urban accessibility.

Urban mobility is inherently multi-modal, with residents relying on complex trip chains that combine walking, cycling, and vehicular travel. Emergency response infrastructure planned around a single, dominant mode creates networks that are systematically fragile and exclusionary to those without access to that mode. Our optimization framework turns this challenge into a strategic asset. By internalizing multiple transportation modes, the model identifies priority locations that perform well across multiple mobility scenarios. Some of these locations may coincide with major transport corridors or interchange areas, but this is a *post-hoc* qualitative interpretation rather than an optimization input. Prioritizing AED deployment at these locations provides a broader baseline level of accessibility across different mobility scenarios, thereby building greater redundancy and spatial balance into the cardiac arrest response system.

Extant literature, particularly in healthcare accessibility research, has convincingly documented that ignoring transportation heterogeneity leads to a misdiagnosis of service coverage and equity. The contribution of this study is to advance this critique from the realm of measurement into the realm of prescriptive planning. We move beyond demonstrating the bias inherent in single-mode location models to developing an optimization framework that helps reduce spatial accessibility disparities. By embedding multiple transportation modes directly into the computational engine of the coverage models, we generate a novel set of spatial solutions that are explicitly sensitive to differences across mobility scenarios. This represents a shift from descriptive, diagnostic analytics to generative, decision-support modeling for more spatially balanced urban health planning.

These findings translate into a three-tiered policy pathway. First, develop differentiated planning standards. AED deployment guidelines should evolve beyond uniform density or radius-based rules to incorporate local mobility contexts, such as the prevalence of zero-car households, cycling infrastructure, and public transit coverage. Second, adopt a phased implementation strategy. In the short term, public health agencies should focus on deploying AEDs at the identified priority sites that perform well across multiple mobility scenarios to achieve rapid, high-impact coverage gains. For the long term, the full multi-modal coverage blueprint should be integrated into municipal master plans and capital improvement programs, ensuring AED siting is coordinated with future transport and urban development projects. Third, foster institutional collaboration. Effective execution requires breaking down silos between public health, transportation, urban planning, and emergency management departments to co-locate AEDs with public transit stations, pedestrian zones, and other critical infrastructure, maximizing visibility and synergy.

Several limitations warrant acknowledgment. First, the analysis employed static network speeds, not accounting for real-time traffic congestion, signal delays, or weather conditions, which may overestimate practical accessibility, especially during peak hours. Second, the model simplified travel behavior, assigning populations to fixed primary modes and neglecting the complexity of emergency trip chains (e.g., drive-park-run) or behavioral uncertainty. Third, demand was modeled using nighttime residential population data, failing to capture the significant diurnal population shifts caused by commuting, potentially leaving employment centers under-protected during daytime hours. Fourth, while an advancement, the “Potential Demand” metric could be refined further with more granular, individual-level health risk data. Fifth, network travel time does not include vertical or indoor access within buildings (e.g., elevators, stairs, corridors), which may add non-negligible delay in high-rise settings and systematically overestimate coverage in dense high-rise districts. Integrating building-height and floor-level data to estimate vertical retrieval impedance—potentially treating AEDs in multi-story buildings as floor-specific facilities rather than single points—represents an important direction for future research. Sixth, using 1 km × 1 km grid centroids may introduce spatial aggregation error, particularly for walking; future work should test finer spatial resolutions such as 100 m or 250 m. Seventh, the driving results are based on assigned network speeds and were not sensitivity-tested under lower peak-period operating speeds; future work should evaluate peak/off-peak or observed traffic speeds where available. Eighth, the mode scenarios are system-level planning assumptions and do not identify which population groups actually have access to a car, bicycle, or other mobility option. Related emergency-resource planning research has explicitly represented heterogeneous mobility characteristics at the population-group level (46). Following this logic, future AED models could assign different feasible travel-mode sets, travel speeds, accessibility constraints, or service requirements to different population groups rather than applying a single system-wide mobility scenario to all residents.

Building on this work, future research should pursue more dynamic, behaviorally nuanced, and technologically advanced modeling avenues. First, integrating Agent-Based Modeling (ABM) could simulate individual spatiotemporal movements and decision-making during cardiac emergencies, leading to location models with greater behavioral realism. Second, future optimization frameworks could explicitly incorporate social equity as a formal objective, designing algorithms that directly minimize access disparities for pre-defined vulnerable subgroups during the optimization process itself. Third, the logic of the model can be extended to plan next-generation response systems by exploring the integration of AED-equipped drones into a hybrid “fixed-point + airborne” delivery network, overcoming ground-based access barriers in congested or remote areas and enhancing overall system resilience. Collaborative vehicle-drone routing frameworks provide a useful methodological analog for coordinating fixed facilities, launch/retrieval locations, and airborne delivery decisions (47).

## 6 Conclusion

This study moves beyond conventional, mono-modal facility location planning by introducing a comprehensive multi-modal optimization framework. By combining an age-risk-weighted demand surface with alternative travel-mode scenarios, we show how AED deployment priorities and coverage estimates change with mobility assumptions and how phased optimization can improve immediate coverage gains. The methodology and the specific case study results provide urban planners and public health officials in Wuhan and similar metropolises with a scientifically grounded, practical tool for supporting more resilient and accessible urban emergency-response systems across different mobility scenarios.

## Data Availability

The original contributions presented in the study are included in the article/supplementary material, further inquiries can be directed to the corresponding author/s.
